# Supplementary material for: SKF-96365 Expels Tyrosine Kinase Inhibitor-Treated CML Stem and Progenitor Cells from the HS27A Stromal Cell Niche in a RhoA-Dependent Mechanism
Source: Cancers (Basel). 2024 Aug 8;16(16):2791. doi: 10.3390/cancers16162791 (PMC11352811; doi:10.3390/cancers16162791)
Supplement: Supplementary file 1 [file cancers-16-02791-s001.zip › cancers-3050597-supplementary/cancers-3050597-supplementary.pdf]

Supplemental Figures

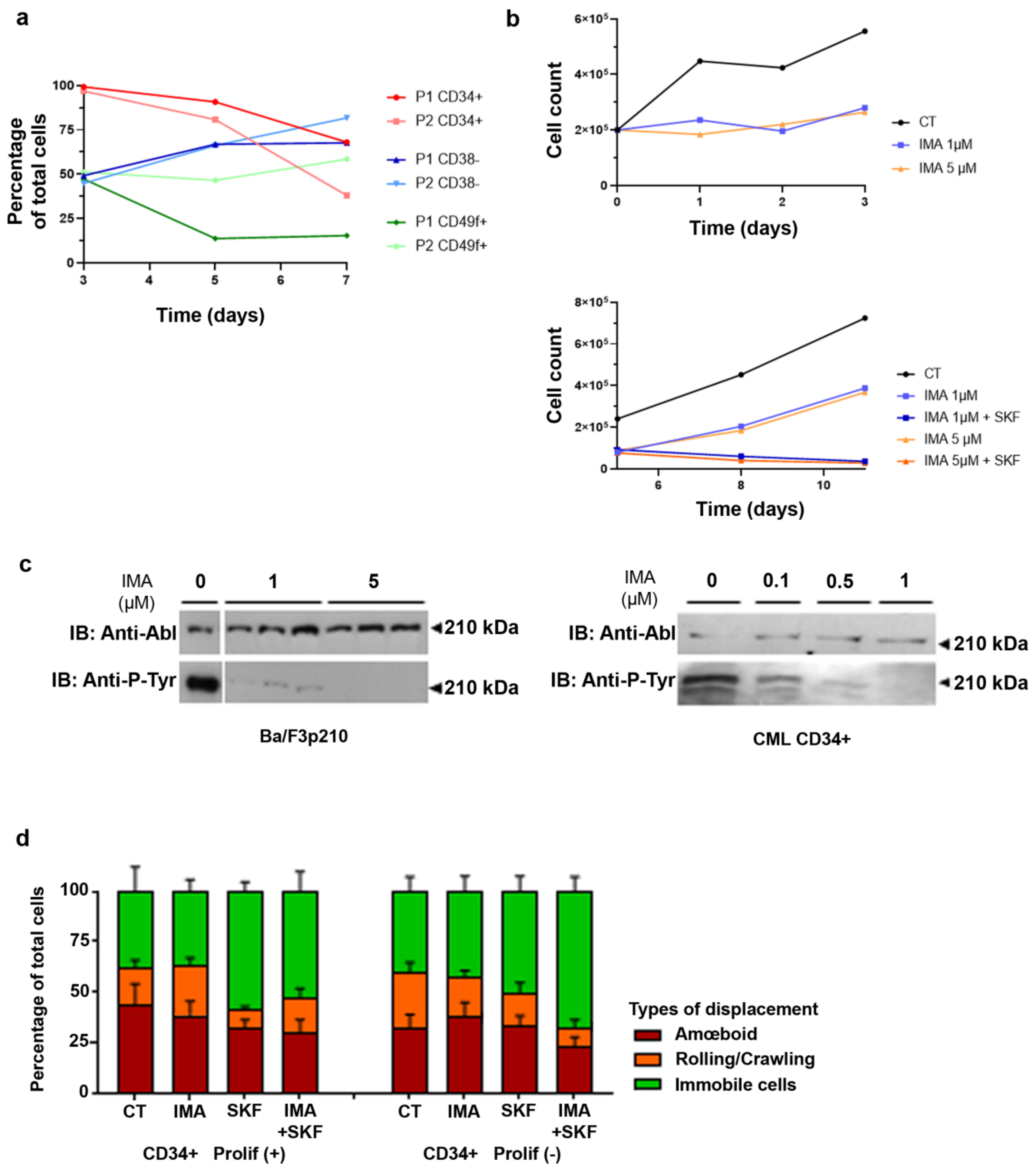

Supplemental Figure S1. (**a**) Persistence of HSC markers during time. (**b**) CML Patient CD34+ cell proliferation under the different conditions (imatinib 1 or 5 µM, +/- SKF-96365). (**c**) Phosphorylation state of Bcr-Abl under imatinib conditions. Western blot analysis of phosphorylated and total p210BCR-ABL during increasing imatinib treatment in Ba/F3p210 cells (left) and CML patient CD34+ cells (right). (**d**) 0.5 × 10^5 CD34+ cells from CML patients, CFSE-sorted as described in Figure 1a (left: prolif +; right: prolif-), were included in 2.5 mg/mL liquid Matrigel diluted in culture medium and incubated with 1 µM imatinib. Each type of displacement (immobile cells, rolling/crawling, or amœboid displacement) was determined through time-lapse videomicroscopy analysis (n > 3).

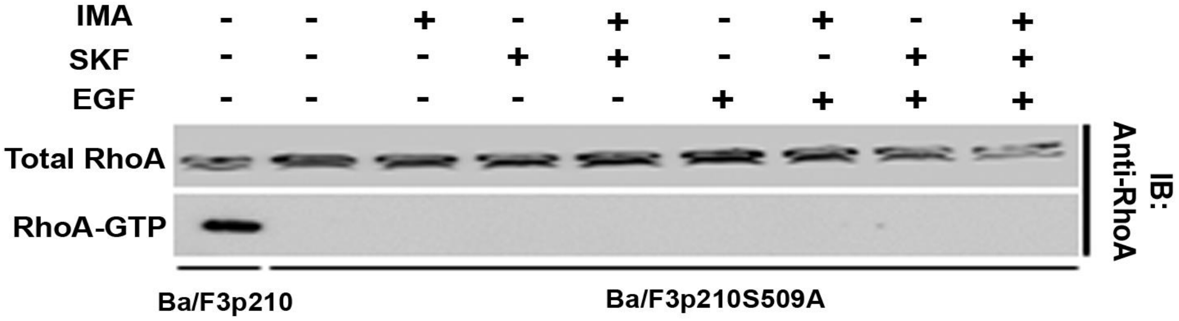

Supplemental Figure S2. Western blot analysis of activated (GTP-RhoA) and total RhoA in Ba/F3p210S509A cells obtained via GST-pulldown. Control experiment using Ba/F3p210 cells is provided.

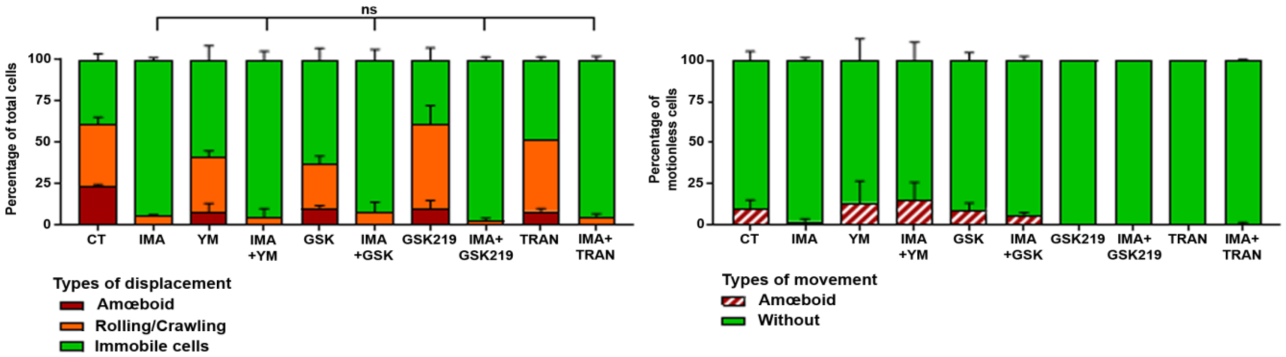

Supplemental Figure S3. Cells (0.5 × 10^5 Ba/F3p210) were included in 2.5 mg/mL liquid Matrigel diluted in culture medium and incubated with or without 5 μM imatinib and/or 10 μM YM-58483 (YM), 10 μM GSK-7975A (GSK), 400 nM GSK2193874 (GSK219), or 100 μM Tranilast (TRAN) for 4 h. Each type of displacement (immobile cells, rolling/crawling, or amœboid displacement) was determined through time-lapse videomicroscopy analysis. Right panel shows the analysis of amoeboid contractions in immobile cells (n > 3). ns: non-significant.

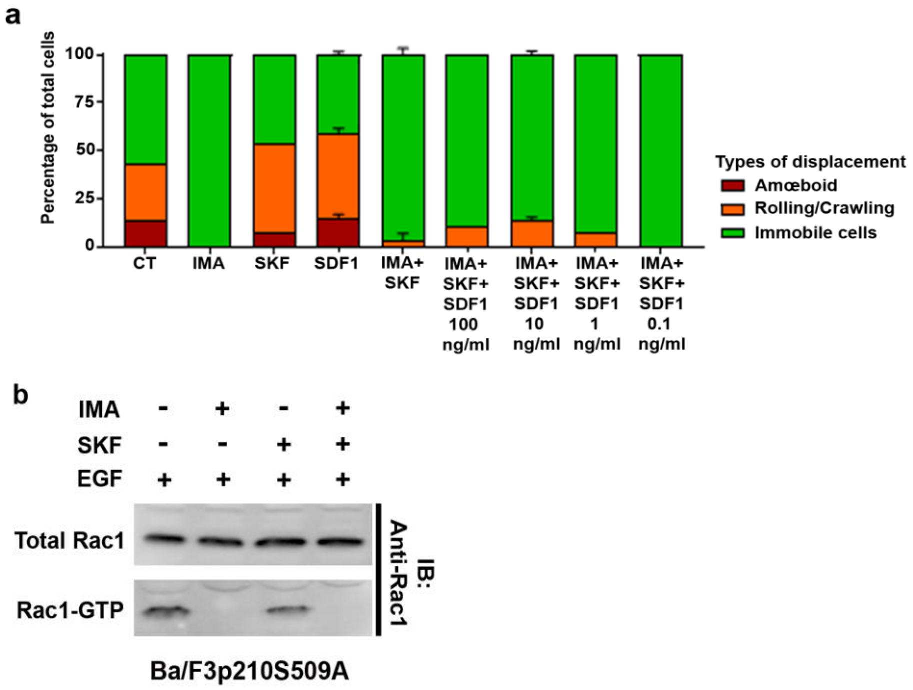

Supplemental Figure S4. (**a**) Cells (0.5 × 10^5 Ba/F3p210 and Ba/F3p210S509A) were included in 2.5 mg/mL liquid Matrigel diluted in culture medium and incubated with or without 5 μM imatinib and/or 40 μM SKF-96365

and/or SDF-1 in increasing concentrations (as indicated). Each type of displacement (immobile cells, rolling/crawling, or amœboid displacement) was determined through time-lapse videomicroscopy analysis (n > 3). (**b**) Western blot analysis of activated (GTP-Rac1) and total Rac1 in Ba/F3p210S509A cells.

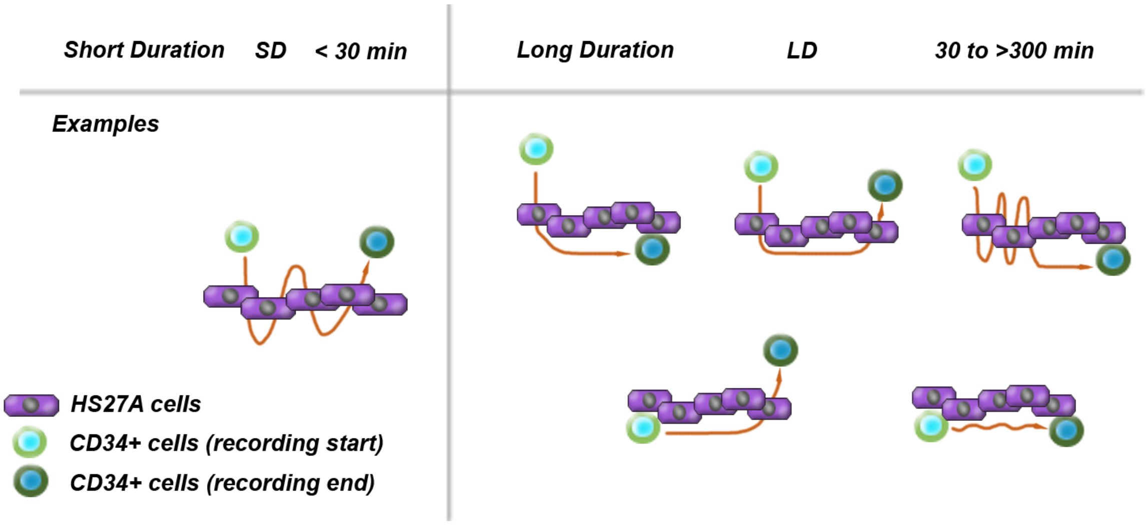

Supplemental Figure S5. Schematic description of CML patient CD34+ cell behavior around HS27A stromal cells during videomicroscopy recordings (light green: recording starts; dark green: recording ends).

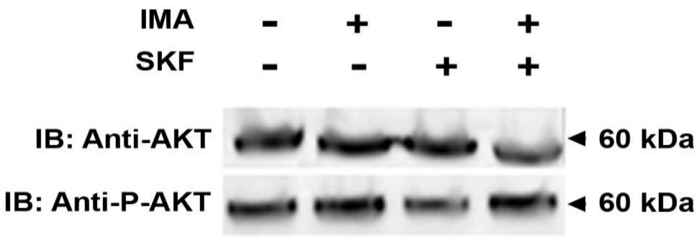

Supplemental Figure S6. Western blot analysis of phosphorylated and total AKT in Ba/F3p210 cells during imatinib and SKF-96365 treatment.
